# Hippocampal Resting-State Functional Connectivity Patterns are More Closely Associated with Severity of Subjective Memory Decline than Whole Hippocampal and Subfield Volumes

**DOI:** 10.1093/texcom/tgaa019

**Published:** 2020-05-28

**Authors:** Lauren Zajac, Bang-Bon Koo, Yorghos Tripodis, Asim Mian, Eric Steinberg, Jesse Mez, Michael L Alosco, Anna Cervantes-Arslanian, Robert Stern, Ronald Killiany

**Affiliations:** Department of Anatomy & Neurobiology, Boston University School of Medicine, Boston, MA 02118, USA; Center for Biomedical Imaging, Boston University School of Medicine, Boston, MA 02118, USA; Department of Anatomy & Neurobiology, Boston University School of Medicine, Boston, MA 02118, USA; Boston University Alzheimer’s Disease Center, Boston University School of Medicine, Boston, MA 02118, USA; Boston University School of Public Health, Boston, MA 02118, USA; Department of Radiology, Boston University School of Medicine, Boston, MA 02118, USA; Boston University Alzheimer’s Disease Center, Boston University School of Medicine, Boston, MA 02118, USA; Department of Neurology, Boston University School of Medicine, Boston, MA 02118, USA; Boston University Alzheimer’s Disease Center, Boston University School of Medicine, Boston, MA 02118, USA; Department of Neurology, Boston University School of Medicine, Boston, MA 02118, USA; Boston University Alzheimer’s Disease Center, Boston University School of Medicine, Boston, MA 02118, USA; Department of Neurology, Boston University School of Medicine, Boston, MA 02118, USA; Department of Neurology, Boston University School of Medicine, Boston, MA 02118, USA; Department of Anatomy & Neurobiology, Boston University School of Medicine, Boston, MA 02118, USA; Boston University Alzheimer’s Disease Center, Boston University School of Medicine, Boston, MA 02118, USA; Department of Neurology, Boston University School of Medicine, Boston, MA 02118, USA; Department of Neurosurgery, Boston University School of Medicine, Boston, MA 02118, USA; Department of Anatomy & Neurobiology, Boston University School of Medicine, Boston, MA 02118, USA; Center for Biomedical Imaging, Boston University School of Medicine, Boston, MA 02118, USA; Boston University Alzheimer’s Disease Center, Boston University School of Medicine, Boston, MA 02118, USA; Boston University School of Public Health, Boston, MA 02118, USA; Department of Neurology, Boston University School of Medicine, Boston, MA 02118, USA

**Keywords:** aging, brain connectomics, cognition, hippocampus, humans, magnetic resonance imaging

## Abstract

The goal of this study was to examine whether hippocampal volume or resting-state functional connectivity (rsFC) patterns are associated with subjective memory decline (SMD) in cognitively normal aged adults. Magnetic resonance imaging data from 53 participants (mean age: 71.9 years) of the Boston University Alzheimer’s Disease Center registry were used in this cross-sectional study. Separate analyses treating SMD as a binary and continuous variable were performed. Subfield volumes were generated using FreeSurfer v6.0, and rsFC strength between the head and body of the hippocampus and the rest of the brain was calculated. Decreased left whole hippocampal volume and weaker rsFC strength between the right body of the hippocampus and the default mode network (DMN) were found in SMD+. Cognitive Change Index score was not correlated with volumetric measures but was inversely correlated with rsFC strength between the right body of the hippocampus and 6 brain networks, including the DMN, task control, and attentional networks. These findings suggest that hippocampal rsFC patterns reflect the current state of SMD in cognitively normal adults and may reflect subtle memory changes that standard neuropsychological tests are unable to capture.

## Introduction

It is important to reliably identify individuals with significant risk of developing dementia in the cognitively normal population in order to test the efficacy of interventions to stop or slow progression before substantial, irreversible neurodegeneration takes place (Sperling et al. 2011a, 2011b). Subjective cognitive decline (SCD), or the self-perception of decline in cognition despite normal neuropsychological test scores, is a potentially valuable measure relevant to the risk of future pathologic objective cognitive decline (Jessen, Amariglio et al. 2014). Cognitively normal individuals reporting SCD, particularly those who specifically report subjective memory decline (SMD), are more likely to have abnormal levels of Alzheimer’s disease (AD) biomarkers and are at increased risk of developing AD dementia (Barnes et al. 2006; Mosconi et al. 2008; Kryscio et al. 2014; Mitchell et al. 2014; Jessen, Wolfsgruber et al. 2014; Amariglio et al. 2015; Buckley et al. 2016; Norton et al. 2017; Tsutsumimoto et al. 2017; Vannini et al. 2017). Despite this, SMD alone is not specific enough to reliably predict future pathologic objective cognitive decline largely because it is a state that can result from varied etiology (Montejo et al. 2011; Jessen, Amariglio et al. 2014). The ease with which SMD data can be collected and evidence supporting that those who endorse it are at greater risk for dementia have resulted in an increased focus on research into SMD and how reports of this state are managed in clinical practice (Jenkins et al. 2015; Rabin et al. 2017).

A better understanding of the neural systems underlying the state of SMD is warranted given the increased research focus on this topic, potential clinical relevance, varied etiology, and lack of sensitivity of neuropsychological tests to this state. Magnetic resonance imaging (MRI) is a noninvasive, widely available, and versatile imaging modality that has been used to study SMD, and MRI measures have been shown to be sensitive to it (Saykin et al. 2006; Hafkemeijer et al. 2013; Wang et al. 2013; Yasuno et al. 2015; Cantero et al. 2016; Ryu et al. 2017; Kawagoe et al. 2019; Viviano et al. 2019). Studies that have assessed differences in brain volume between those who report SMD and those who do not have consistently reported decreased hippocampal volume in individuals who report SMD (van der Flier et al. 2004; Saykin et al. 2006; Striepens et al. 2010; Hafkemeijer et al. 2013; Cantero et al. 2016). SMD was treated as a binary factor in most of these studies, thus it is unclear whether whole hippocampal or subfield volumes are associated with the severity of SMD or whether those with smaller hippocampal volumes are more likely to report SMD as they age. The handful of studies examining resting-state functional connectivity (rsFC) patterns in cognitively normal individuals with SMD have reported mixed results; most have similarly treated SMD as binary. Some rsFC studies have found stronger rsFC in those reporting SMD (Hafkemeijer et al. 2013; Kawagoe et al. 2019), whereas others have found weaker rsFC in those reporting SMD (Wang et al. 2013; Yasuno et al. 2015; Viviano et al. 2019). Some of these studies have focused on the default mode network (DMN), which is heavily associated with memory function and self-referential processing and affected in aging and AD (see Buckner et al. 2005 for review; Andrews-Hanna et al. 2007, 2010; Jones et al. 2011, 2015). However, these rsFC studies do not focus on rsFC patterns between the hippocampus and the rest of the brain despite the central role of the hippocampus in memory (Squire 1992) and AD (Jack et al. 1992, 1999), and the reported effects of SMD on hippocampal volume. This, in addition to the small number of studies and mixed direction of findings, calls for additional study of the rsFC patterns associated with SMD in order to further our understanding of how the state of SMD is represented in the brain and the specifics of hippocampal involvement in this state. The diversity of neuroimaging measures that can be derived from MRI data makes it a promising tool for gaining a better understanding of the state of SMD and determining the types of MRI measures that are most closely associated with SMD. Furthermore, these aspects of MRI may eventually help pinpoint more reliable imaging biomarkers for differentiating between those in the cognitively normal population who express features in the brain that are early signs of dementia from those who express features solely related to factors such as mood and medication, both of which can impact memory.

Our overarching goal in this cross-sectional study was to assess the patterns of hippocampal volume and interactions between the hippocampus and the rest of the brain that are closely associated with the presence and severity of SMD in the same sample of cognitively normal individuals. Our hypothesis was that individuals reporting SMD would have smaller hippocampal volumes and differential rsFC between the hippocampus and the rest of the brain, particularly the DMN, due to the central role of the hippocampus and DMN in memory. To test this, we treated SMD as a binary and a continuous variable in separate analyses. The first set of analyses separated our cognitively normal sample into 2 groups: those reporting elevated levels of SMD (SMD+) and those not reporting SMD (SMD−). The second set treated SMD as a continuous variable, allowing us to examine the hippocampal volumetric and rsFC variables most closely associated with the severity of reported SMD. Treating SMD as both binary and continuous is important to effectively interpret our results in the context of the existing literature (much of which has considered SMD as binary) while also providing new information on hippocampal features associated with the severity of perceived memory decline.

## Materials and Methods

### Participant Demographics, Clinical and Neuropsychological Assessment, and Definition of SMD

Data were obtained from the research registry of the Boston University Alzheimer’s Disease Center (BUADC). The BUADC is one of 32 centers (nia.nih.gov/health/alzhiemers-disease-research-centers) funded by the National Institute on Aging and contributes data to the National Alzheimer’s Coordinating Center. A description of the registry is provided elsewhere (Galetta et al. 2017). All participants received a consensus diagnosis of cognitively normal through the evaluation of neuropsychological scores and clinical/health information. Participants completed a number of cognitive measures, including the Mini-Mental State Examination (MMSE), the List Learning test from the Neuropsychological Assessment Battery (NAB) (Stern and White 2003), Trailmaking Test Part B, the short form of the Geriatric Depression Scale (GDS) and some participants underwent apolipoprotein E (APOE) genotyping. Cognitively normal participants with MRI scans were selected for this study, which resulted in data from 53 participants being used. The collection of these data was approved by the institutional review board at the Boston University School of Medicine and conducted in accordance with the Helsinki Declaration. All participants gave written informed consent.

The Cognitive Change Index (CCI) is a 20-item scale of cognitive ability that asks individuals whether they feel their ability in multiple cognitive domains has been stable or decreased over the past 5 years (Rattanabannakit et al. 2016). We used the first 12, memory-based items of the CCI to separate our participants into SMD+ and SMD− in our first set of analyses and as a measure of the severity of SMD in our second set of analyses. All references to CCI score in this paper refer to participants’ responses to these first 12 items at a single time point. BUADC participants complete the CCI at each yearly visit; the CCI score closest to the date of each participant’s MRI scan was used. Participants were classified as SMD+ if their score on these 12 CCI items totaled 16 or greater (Aisen et al. 2015), resulting in 29 SMD+ and 24 SMD− participants. Demographic information on these groups is shown in Table 1. In analyses treating SMD as continuous, participants’ total score on the same 12 CCI items represented the severity of SMD with higher scores representing stronger perception of memory decline.

**Table 1:** Sample demographics

Demographic variable	Mean (Standard deviation)	
	SMD− (*n* = 24)	SMD+ (*n* = 29)
Age (years)	72.1 (±10.4)	71.8 (±6.04)
Sex (male/female)	9/15	10/19
Education (years)	15.5 (±2.67)	16.7 (±1.93)
Resting-state fMRI mean absolute displacement	0.246 (±0.183)	0.299 (±0.188)
Resting-state fMRI mean relative displacement	0.191 (±0.090)	0.215 (±0.104)
APOE ε4 alleles (number of participants with at least one allele/number of participants with APOE data in group)	5/20	8/19
APOE ε2 alleles (number of participants with at least one allele/number of participants with APOE data in group)	4/20	1/19
CCI (12 memory items only)	13.4 (±1.1)	23.4 (±7.07)^**^
MMSE	29.0 (±1.02)	29.1 (±0.860)
NAB List Learning short delay (raw)	8.79 (±2.25)	8.41 (±2.23)
NAB List Learning long delay (raw)	8.54 (±2.17)	8.41 (±1.97)
NAB retention (raw)	0.941 (±0.0902)	0.931 (±0.0972)
Trailmaking test Part B (raw, seconds)	72.5 (±27.6)	75.4 (±24.5)
GDS	0.458 (±0.977)	0.966 (±1.15)^*^

Notes: The mean and standard deviation of each variable is listed for SMD− and SMD+. For sex, the number of males and females per group is listed. For APOE alleles, the number of participants with at least one APOE ε4 or ε2 allele per group is listed over the number of participants for which APOE data were available in each group. APOE data was not available for 4 SMD− participants and 10 SMD+ participants. Only one participant had 2 APOE ε4 alleles, and this participant was in the SMD+ group. ^*^P < 0.05, ^**^P < 0.0001.

### Magnetic Resonance Imaging

T1-weighted (T1W) and resting-state functional MRI (rsfMRI) data were collected at the Center for Biomedical Imaging at the Boston University School of Medicine on a Philips 3T Achieva scanner (Best, the Netherlands) using a 32-channel head coil. T1W magnetization prepared rapid gradient echo (MP-RAGE) sagittal images were acquired (repetition time/echo time [TR/TE] = 6.7/3.1 ms; acquisition matrix = 256 × 254, 150 slices; field of view [FOV] = 250 × 250 × 180 mm; voxel size = 0.98 × 0.98 × 1.2 mm; flip angle = 9°). Although rsfMRI data were collected, participants were asked to stay awake, fixate on a white dot and let their minds wander for 10 min. The rsfMRI data consisted of T2^*^-weighted axial images with blood oxygenation level dependent contrast (TR/TE = 3000/30 ms; acquisition matrix = 64 × 59, 48 slices; FOV = 212 × 198.75 × 159 mm; voxel size = 3.31 × 3.31 × 3.31 mm; echo planar imaging [EPI] factor = 59).

### Image Processing

All MRI scans were converted from digital imaging and communications in medicine (DICOM) to neuroimaging informatics technology initiative format (NIfTI) using dcm2nii and visually inspected for artifacts before use. Data from participants whose rsfMRI data contained motion spikes greater than 3 mm were not used.

### Hippocampal Subfields

**Figure 1:**
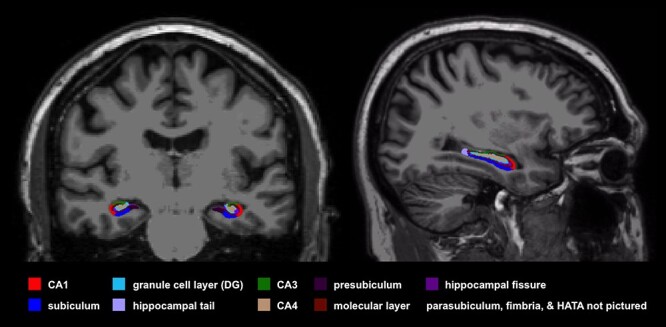
A representative hippocampal subfield segmentation from one participant is shown. Subfield segmentations were performed in FreeSurfer v6.0, which divides the hippocampus into 12 subfields in each hemisphere. Most of the subfields are shown in this image, with the parasubiculum, fimbria, and hippocampal-amygdala transition area (HATA) not pictured. All subfields volumes were adjusted for eTIV and age.

T1W images were processed with the recon-all pipeline, including automated hippocampal subfield segmentation (Iglesias et al. 2015) in FreeSurfer v6.0 (https://surfer.nmr.mgh.harvard.edu); subfields were visually inspected to ensure no gross errors in segmentation were present. None were present and no manual edits were made to the segmentation maps. Subfield volumes, whole hippocampal volumes, and estimated total intracranial volume (eTIV) were extracted. Figure 1 shows a representative hippocampal subfield segmentation. A total of 26 hippocampal volumetric measures were extracted: left/right whole hippocampal volume and 12 subfields from each hemisphere. All hippocampal volumetric measures were adjusted for age and eTIV using a regression model built from the data of all 53 participants. Age- and eTIV-adjusted volumes were used in all analyses.

### Hippocampal rsFC

A single-session independent components analysis was run on each participant’s rsfMRI data using MELODIC v3.14 (Filippini et al. 2009) in FSL v5.08 (Jenkinson et al. 2012). The first 10 volumes of each scan were discarded. Preprocessing included highpass filtering (cutoff = 100 s), motion correction (MCFLIRT) and spatial smoothing (full width half maximum [FWHM] = 5 mm). Timecourses were variance-normalized and automatic dimensionality estimation was performed. The preprocessed data output from MELODIC were entered into FSL’s FIX v1.06 (Griffanti et al. 2014; Salimi-Khorshidi et al. 2014) and processed using the Standard.RData trained-weights file, a threshold of 20 and additional motion cleanup. FIX automatically classifies the components that constitute the output of MELODIC as noise or signal. In rsfMRI analyses, it is particularly important to account for components of the signal reflecting noise due to motion, susceptibility, cardiac pulsations, white matter, and cerebrospinal fluid pulsations. A single viewer (Dr Zajac) visually inspected all components to ensure the accuracy of FIX component classification, as is recommended when FIX is run using the standard training data. Components that the viewer determined to be misclassified as signal or noise based on visual inspection of the spatial pattern, the timecourse, and the frequency composition of the component were accurately relabeled as signal or noise. The components classified as noise were then regressed out of the rsfMRI data. Nonlinear registration with a warp resolution of 2 mm was used to transform data into MNI152 2 mm space. All registrations were visually inspected to ensure accurate alignment with the MNI template.

The rsFC strength was calculated between the head and body of the hippocampus and 264 spherical regions of interest (ROIs) defined by Power et al. (2011), which will be referred to as the Power ROIs throughout. To create hippocampal head and body ROIs, hippocampal maps generated in the recon-all pipeline in FreeSurfer v6.0 were divided into head, body, and tail using the method described in Greene and Killiany (2012). Each participant’s left or right hippocampal ROI (defined in FreeSurfer’s recon-all pipeline) was loaded into Freeview and viewed in the sagittal plane. To define the boundary between the head and body of the hippocampus, the editor (Dr Zajac) first advanced medially to the last slice where the head and the body of the hippocampal ROI were continuous (i.e., connected by ROI voxels). The most narrow point between the head and the body that did not include any portion of the head was selected with the cursor and then viewed in the coronal plane. This was defined as the first slice of the hippocampal body ROI; all coronal slices anterior to the hippocampal ROI were defined as the hippocampal head ROI. To define the boundary between the body and the tail of the hippocampus, the editor advanced posteriorly through coronal sections until the first section in which the fimbria of the fornix was fully evident. This was defined as the first slice of the hippocampal tail. All coronal slices anterior to this slice and posterior to the head-body hippocampal boundary as defined above were defined as the hippocampal body ROI. This process was carried out for the left and the right hippocampus separately. Head and body hippocampal labels from each hemisphere were extracted as volumes in each participant’s anatomical space, eroded by one voxel to prevent overlap, and nonlinearly transformed into MNI152 2 mm space. The accuracy of each transformation was ensured. To create the Power ROIs, binary, spherical regions with a 10 mm diameter were centered at the MNI coordinates corresponding to the 264 Power ROIs. The coordinates of these ROIs were obtained from the supplementary information associated with Power et al. (2011) and network assignments for each ROI were obtained from www.jonathanpower.net. The Power ROIs are categorized into the following 13 networks: somatomotor network (SOM), auditory network (AUD), visual network (VIS), DMN, memory retrieval network (MRN), dorsal attention network (DAN), ventral attention network (VAN), salience network (SN), cingulo-opercular task control network (COTCN), frontoparietal task control network (FPTCN), subcortical network (SUB), cerebellar network (CER) and uncertain.

**Figure 2:**
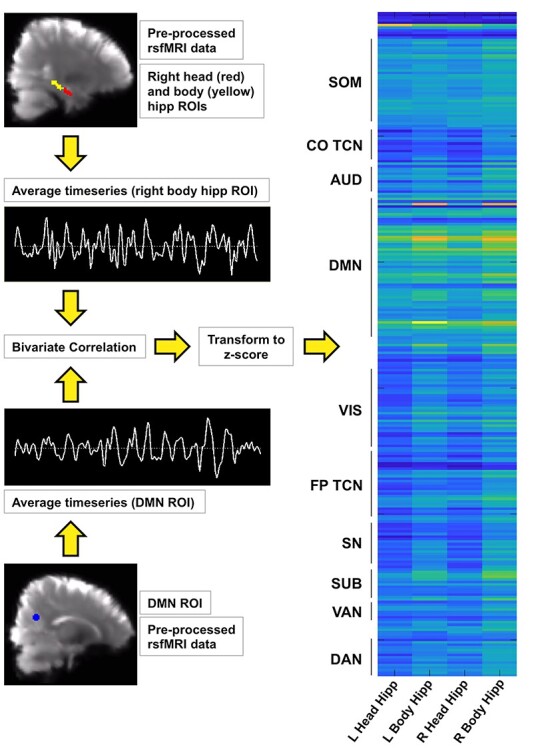
A schematic representing hippocampal rsFC strength calculations is shown. Top left: representative RHeadHipp (red) and body of the hippocampus (yellow) ROIs are shown overlaid on preprocessed rsfMRI data in MNI 2 mm space. The hippocampal mask generated in FreeSurfer v6.0 was divided into head, body, and tail to create the head and body ROIs. Bottom left: a Power ROI that belongs to the DMN is shown overlaid on preprocessed rsfMRI data in MNI 2 mm space. Each Power ROI was a 10 mm-diameter binary sphere, and there were 264 Power ROIs in total. For each participant, the average band-pass filtered blood oxygenation level-dependent timeseries from the right and left head and body of the hippocampus and each Power ROI was extracted. Examples are shown in this image. Bivariate correlation was run between each hippocampal ROI timeseries and each Power ROI timeseries, transformed to *z*-scores and assembled into a 4 × 264 rsFC matrix (right). A representative rsFC matrix from one participant is shown, in which yellow represents strong rsFC and dark blue represents weak rsFC. The Power ROIs are largely grouped by brain network, and these are shown to the left of the matrix.

The rsFC strength between hippocampal and Power ROIs was calculated using CONN toolbox v18.a (Whitfield-Gabrieli and Nieto-Castanon 2012) running in MATLAB R2017a. Each participant’s preprocessed rsfMRI data were band-pass filtered (0.01–0.1 Hz). Bivariate correlations between the head and body of the left and right hippocampus and Power ROIs were performed and transformed into *z*-scores, which yielded a 4 × 264 hippocampal rsFC matrix for each participant. The *z*-score in each cell of these matrices represents the functional connectivity strength between the head or body of the left or right hippocampus and another region of the brain. Figure 2 displays a schematic summarizing these rsFC calculations.

### Statistics

#### Demographic Variables

The following analyses were performed in JMP Pro v13. Assessing whether the SMD+ and SMD− groups differed in factors other than the degree of reported SMD was important to our analyses treating SMD as binary. Continuous demographic variables were compared between groups using two-tailed *t*-tests (age) or Wilcoxon tests (education, CCI score, GDS, MMSE, and rsfMRI motion). Nominal demographic variables (sex, APOE ε2 and ε4 alleles) were compared using chi-square tests. The effect of group on NAB List Learning (short delay, long delay and retention) and Trailmaking Test Part B raw scores was tested with age, education and sex as covariates. For these comparisons, *P* values of less than 0.05 were considered to be statistically significant.

Relevant to our analyses treating SMD as continuous, we assessed whether any demographic variables (age, education, sex, APOE ε4, APOE 2, MMSE, and GDS) were significantly associated with CCI score. Demographic variables that showed a significant relationship with CCI score were assessed in greater detail with respect to volumetric and rsFC results, and these analyses are described in the Results section. We also assessed whether NAB List Learning (short delay, long delay, and retention) and Trailmaking Test Part B raw scores were associated with CCI score, and age, education and sex were included as covariates. For these analyses, *P* values of less than 0.05 were considered to be statistically significant.

#### Hippocampal Volume Between-Group Analyses

One-tailed *t*-tests were performed predicting smaller age- and eTIV-adjusted whole hippocampal and subfield volumes in individuals with SMD+. First, whole hippocampal volumes were tested. For these tests, *P* values of less than 0.05 were considered statistically significant. We then assessed whether SMD effects were present in specific subfields. false discovery rate (FDR)-adjusted *P* values were calculated for the set of subfield volume tests (Yekutieli and Benjamini 1999), and FDR-adjusted *P* values less than 0.05 were considered statistically significant.

#### Hippocampal rsFC Strength Between-Group Analyses

Two-tailed *t*-tests were performed to identify differential connections between the left head, left body, right head and right body of the hippocampus and the 264 Power ROIs (264 connections associated with each ROI, 1056 connections total). Due to the large number of connections, we assessed the significance of “patterns” of differential connectivity in 2 ways. The first way focused on the number of differential connections per hippocampal ROI. We examined this to assess whether the connectivity between one hippocampal region and the rest of the brain was affected more strongly than the other hippocampal regions in SMD+. Once we performed the *t*-tests and visualized differential connections, it was clear that the number of differential connections between the right body of the hippocampus and the Power ROIs was much larger than the number of differential connections between the other 3 hippocampal ROIs and the Power ROIs. Furthermore, all but one differential connection were weaker in SMD+ relative to SMD−, so we only considered differences in this direction moving forward. To test whether the number of weaker connections between the right body of the hippocampus and the rest of the brain in SMD+ was significantly greater than the number of weaker connections between the other hippocampal ROIs and the rest of the brain, we created a difference score [rsFC difference score = (# right body hippocampus conns significantly weaker in SMD+) – (sum of the # of left head, left body and right head hippocampus conns significantly weaker in SMD+)]. Group labels were randomly permuted 10 000 times to create a distribution of difference scores, and we tested whether our difference score was greater than chance (*P* = 0.05). For this analysis, a *P* value of less than 0.05 was considered to be statistically significant. Permutation analyses were performed in MATLAB R2017a.

The second way we assessed differential connectivity was adapted from the contingency network analysis approach described in Sripada et al. (2014) and recently applied in the context of SCD in Contreras et al. (2019). Briefly, this approach uses permutation tests to determine whether the number of differential connections per resting-state network is greater than chance. We applied this within the set of differential connections involving the right body of the hippocampus and tested whether the number of weaker connections between this region and any of the 13 resting-state networks into which the Power ROIs are classified was greater than chance. We randomly permuted the group labels 10 000 times to create a distribution of the number of weaker connections between the right body of the hippocampus and each network. We tested whether the number of weaker connections between the right body of the hippocampus and each network was greater than chance (*P* = 0.05). FDR-adjusted *P* values were calculated for the set of network-specific tests (Yekutieli and Benjamini 1999). For these analyses, FDR-adjusted *P* values less than 0.05 were considered to be statistically significant.

#### Hippocampal Volume and rsFC Strength Correlational Analyses with CCI Score

We performed a similar set of analyses to those described above but treated SMD as a continuous variable. Linear correlations were performed between left and right whole hippocampal and subfield volumes and CCI score. One-tailed tests predicting an inverse relationship between CCI score and age- and eTIV-adjusted whole hippocampal and subfield volumes were performed. For the whole hippocampal volume correlation analyses, P values of less than 0.05 were considered statistically significant. For the set of subfield volume correlation analyses, FDR-adjusted *p* values less than 0.05 were considered to be statistically significant.

Linear correlations with no predicted direction were also performed between CCI score and hippocampal rsFC strength and the 2 approaches described in section Hippocampal rsFC Strength Between-Group Analyses were applied to determine the significance of the analyses. All significant linear correlations were inverse; those with higher CCI scores (i.e., more severe SMD) had weaker rsFC strength between the hippocampus and the rest of the brain. Similar to the between-group analyses, it was clear that a majority of these significant inverse correlations involved interactions between the right body of the hippocampus and Power ROIs. We tested whether the number of connections between the right body of the hippocampus and other brain regions showing an inverse correlation with CCI score was significantly greater than chance using a difference score analogous to that described in section Hippocampal rsFC Strength Between-Group Analyses. For this analysis, a *P* value of less than 0.05 was considered to be statistically significant. Similarly, we assessed network specificity among these inverse relationships between CCI score and rsFC strength. We randomly permuted CCI scores 10 000 times and created a distribution of the number of significant inverse correlations between the right body of the hippocampus and each brain network. We then tested whether the number of significant inverse correlations between CCI score and rsFC strength with each brain network was greater than chance (*P* = 0.05). FDR-adjusted *P* values were calculated for the set of network specific tests (Yekutieli and Benjamini 1999). For these analyses, FDR-adjusted *P* values less than 0.05 were considered to be statistically significant.

#### Hippocampal Volume and rsFC Strength Correlational Analyses with GDS Score

Because the details of the relationship between depressive symptoms (clinical or subclinical), SMD and hippocampal features in aged adults are not fully understood, we conducted an exploratory analysis that examined what patterns of hippocampal FC were correlated with GDS score using the same approach used to carry out the CCI score analyses.

## Results

### Demographic Variables

Most demographic variables did not differ significantly between SMD+ and SMD− (Table 1). By design, memory CCI score was significantly higher in SMD+ (*P* < 0.0001). GDS score was also significantly higher in SMD+ (*P* = 0.0303), which has been reported by others (van der Flier et al. 2004; Montejo et al. 2011; Amariglio et al. 2015; Masters et al. 2015). GDS score reflected subclinical depressive symptoms for all participants, most reported no symptoms at all, [score range = 0–4; score 0 (*n* = 32), score 1 (*n* = 10), score 2 (*n* = 5), score 3 (*n* = 5), score 4 (*n* = 1)] and GDS score differed by an average of one point between groups. We assessed whether this single-point average difference was due to GDS item 10: “Do you feel you have more problems with memory than most?” because a greater number of individuals in the SMD+ group responded yes to this question (7 SMD+ vs. 1 SMD−, *P* = 0.0318). We subtracted the response to this question from participants’ total GDS scores and the group difference in GDS score no longer remained (*P* = 0.121), ultimately reflecting a lack of significant difference in subclinical depressive symptoms between groups that is not related to memory.

Similarly, most demographic variables did not show a significant relationship with memory CCI score (range = 12–41). As expected, there was a significant positive relationship between GDS and CCI score (*P* = 0.0002) that remained, but was weaker, when subtracting the response to GDS item 10 (*P* = 0.0135). For this reason, in addition to the fact that the details of the relationship between depressive symptoms, SMD, and hippocampal features in aged adults are not fully understood, we assessed whether our correlational results remained significant when controlling for GDS score and we performed exploratory analyses between hippocampal rsFC strength and GDS score that were analogous to those performed with CCI score (Supplementary Material). No other demographic variables showed significant relationships with CCI score.

### Hippocampal Volume Between-Group Analyses

Table 2 shows the average age- and eTIV-adjusted whole hippocampal and subfield volumes in each group. Left hippocampal volume was significantly decreased in SMD+ (*P* = 0.0412). Five subfield volumes in the left hemisphere were also significantly decreased in SMD+: tail (*P* = 0.0293), cornu ammonis (CA) 1 (*P* = 0.00875), molecular layer (*P* = 0.0425), CA3 (*P* = 0.0251) and CA4 (*P* = 0.0344). Differences in subfield volumes did not survive correction for multiple comparisons.

**Table 2:** Average age- and eTIV-adjusted whole hippocampal and subfield volumes

Hippocampal regional volume	SMD− average volume (standard deviation)	SMD+ average volume (standard deviation)
Left whole hippocampus	65.0 (±261)	−53.8 (±227)^*^
Left hippocampal tail	17.5 (±62.4)	−14.5 (±57.5)^*^
Left subiculum	1.84 (±42.7)	−1.52(±40.6)
Left CA1	19.1 (±51.8)	−15.9 (±51.6)^**^
Left hippocampal fissure	6.22 (±26.2)	−5.12 (±30.8)
Left presubiculum	−0.504 (±34.4)	0.417 (±35.7)
Left parasubiculum	−0.083 (±13.4)	0.0687 (±12.9)
Left molecular layer	10.9 (±42)	−8.99 (±40.1)^*^
Left granule cell layer/DG	3.93 (±23.6)	−3.25 (±18.6)
Left CA3	7.15 (±27.1)	−5.92 (±18)^*^
Left CA4	5.07 (±20.6)	−4.2 (±15.7)^*^
Left fimbria	−0.714 (±17.7)	0.591 (±17.1)
Left HATA	0.871 (±9.25)	−0.721 (±7.97)
Right whole hippocampus	20.1 (±258)	−16.7 (±214)
Right hippocampal tail	5.54 (±48.4)	−4.59 (±43.8)
Right subiculum	0.879 (±47.7)	−0.727 (±30.2)
Right CA1	2.72 (±66.7)	−2.25 (±55.6)
Right hippocampal fissure	−3.2 (±28.6)	2.65 (±37.5)
Right presubiculum	2.93 (±34.3)	−2.43 (±24.1)
Right parasubiculum	0.293 (±7.6)	−0.242 (±7.69)
Right molecular layer	3.93 (±50)	−3.25 (±40.7)
Right granule cell layer/DG	0.332 (±21.8)	−0.274 (±27.1)
Right CA3	0.54 (±20.4)	−0.447 (±25.6)
Right CA4	1.37 (±18.2)	−1.13 (±22.9)
Right fimbria	1.13 (±18.3)	−0.934 (±14.8)
Right HATA	0.466 (±9.99)	−0.386 (±8.17)

Notes: ^*^P < 0.05, ^**^P < 0.01 (uncorrected).

### Hippocampal rsFC Strength Between-Group Analyses

**Figure 3:**
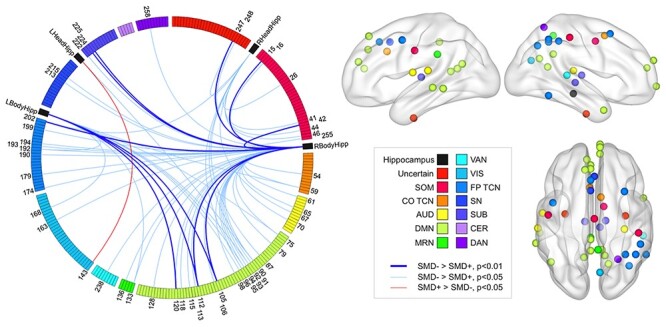
Differential hippocampal rsFC in SMD+ versus SMD−. The 69 hippocampal rsFC variables showing differential connectivity in SMD+ relative to SMD− displayed (left) on a Circos graph (Krzywinski et al. 2009) and a subset of these variables represented (right) on cortical surfaces created in Brain Net Viewer v1.6 (Xia et al. 2013). The colors displayed on the legend represent specific brain networks. The colors of the regions displayed on the circular boundary on the Circos graphs and the spheres on the cortical surfaces correspond to these brain networks. The circular boundary on the Circos graph displays the 264 Power ROIs grouped by the networks shown in the legend. The black regions on the Circos graph represent the left and right head and body of the hippocampus. The red and blue lines on the Circos graph represent connections between hippocampal ROIs and Power ROIs that were significantly stronger in SMD+ and SMD−, respectively. The numbers on the Circos graph correspond to specific Power ROIs whose connectivity strength with any of the hippocampal ROIs differed between groups. The number of connections between the right body of the hippocampus and other brain regions weaker in SMD+ relative to the number of weaker connections between the other hippocampal regions and the rest of the brain combined was greater than chance. Only the nodes showing weaker rsFC strength with the right body of the hippocampus in SMD+ are displayed on the cortical surfaces on the right. The number of weaker connections between the right body of the hippocampus and regions in the DMN (pale green nodes, right) was significantly greater than chance and survived correction for multiple comparisons. LBodyHipp, left body of the hippocampus; LHeadHipp, left head of the hippocampus.

Hippocampal rsFC strength with the rest of the brain was decreased in SMD+; 68 of 69 connections were weaker in SMD+ (Fig. 3). Of the 68 weaker connections, 1 involved the left head, 11 involved the left body and 10 involved the right head of the hippocampus. All of these are below the false positive rate of 13 differential connections per hippocampal region expected for an alpha of 0.05. In contrast, 46 connections involving the right body of the hippocampus were weaker in SMD+. The number of significantly weaker connections involving the right body of the hippocampus relative to the number involving the other 3 hippocampal regions combined was significantly greater than chance (*P* = 0.0071).

We next assessed whether the weaker connections between the right body of the hippocampus and the rest of the brain in SMD+ showed a network-specific pattern. The number of significantly weaker connections between the right body of the hippocampus and the auditory (*P* = 0.0355), default mode (*P* = 0.0038), memory retrieval (*P* = 0.029), frontoparietal task control (*P* = 0.014) and subcortical (*P* = 0.0364) networks were all greater than chance. Only the number of weaker connections between the right body of the hippocampus and the DMN survived multiple comparison correction (*P*_FDR_ = 0.0494).

### Hippocampal Volume and rsFC Strength Correlational Analyses with CCI Score

Neither whole hippocampal nor subfield volumes were significantly correlated with CCI score (*P*’s all greater than 0.05); we did not pursue these analyses further.

**Figure 4:**
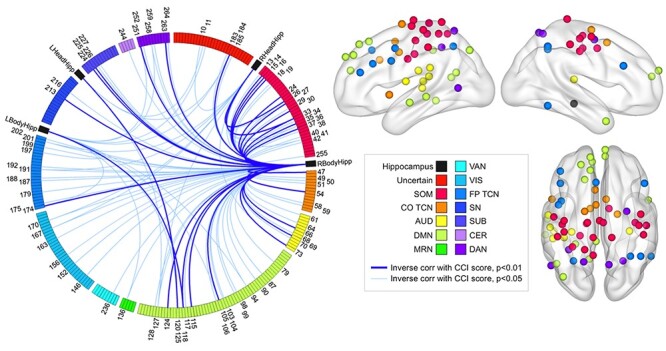
Hippocampal rsFC strength is inversely associated with SMD severity. The 115 connections between hippocampal and Power ROIs showing an inverse relationship with CCI score are displayed on the Circos graph. No positive relationships were found. The number of connections between the right body of the hippocampus and other brain regions showing an inverse relationship with SMD relative to the number of connections between the other hippocampal regions and the rest of the brain showing an inverse relationship with SMD severity combined was greater than chance. The number of connections between the right body of the hippocampus and regions in the SOM, AUD, COTCN, FPTCN, DAN, and DMN inversely associated with SMD severity was significantly greater than chance and survived correction for multiple comparisons. The nodes representing the brain regions within these 6 networks whose connection strength with the right body of the hippocampus showed an inverse relationship with SMD severity are displayed on the right on cortical surfaces created in Brain Net Viewer v1.6 (Xia et al. 2013).

In contrast, CCI score showed significant inverse correlations with rsFC strength between the hippocampus and several brain regions (Fig. 4). All 115 significant correlations were inverse—those with higher CCI score had weaker rsFC strength between the hippocampus and the rest of the brain. Of these 115 relationships, 85 involved the right body of the hippocampus, well above the false positive rate of 13. Of the remaining relationships, 4 involved the left head, 12 involved the left body and 14 involved the RHeadHipp. Similar to the results presented in section Hippocampal rsFC Strength Between-Group Analyses, the number of inverse correlations between CCI score and rsFC strength involving the right body of the hippocampus relative to the number of inverse correlations involving the other 3 hippocampal regions combined was significantly greater than chance (*P* = 0.0007). We assessed whether right hippocampal volume was a mediator of the inverse correlations found between SMD severity and rsFC strength between the right body of the hippocampus and other brain regions. All relationships remained significant when either raw or age and eTIV-adjusted right hippocampal volume was included as a covariate in the analyses.

We next assessed the network specificity of the 85 significant inverse correlations involving the right body of the hippocampus. The number of relationships between CCI score and rsFC strength between the right body of the hippocampus and somatomotor (*P* = 0.0044), cingulo-opercular task control (*P* = 0.0094), auditory (*P* = 0.0041), default mode (*P* = 0.008), frontoparietal task control (*P* = 0.0103), subcortical (*P* = 0.0382) and dorsal attention (*P* = 0.014) networks were significantly greater than chance. All of these remained significant after multiple comparison correction except for specificity related to the subcortical network: somatomotor (*P*_FDR_  = 0.0268), cingulo-opercular task control (*P*_FDR_  = 0.0268), auditory (*P*_FDR_  = 0.0268), default mode (*P*_FDR_  = 0.0268), frontoparietal task control (*P*_FDR_  = 0.0268) and dorsal attention (*P*_FDR_  = 0.0303) networks.

In light of the results discussed above and the significant correlation between GDS and CCI score noted in section Demographic Variables, we examined the correlations between GDS score and hippocampal rsFC strength. First, we assessed whether the rsFC strength of any connections involving the right body of the hippocampus whose strength was significantly correlated with CCI score was significantly correlated with GDS score. Only 10 connections between the right body of the hippocampus and other brain regions showed a significant correlation with GDS score and all of them were positive (those with higher GDS scores had stronger hippocampal connectivity). Only 1 of these 10 connections overlapped with the 85 whose strength was inversely correlated with CCI score. This connection was with the “uncertain” network, which is a collection of ROIs that could not be confidently assigned to any of the other 12 networks. To further verify that GDS score was not associated with our CCI score results, we assessed whether significant relationships between CCI score and rsFC strength of the 85 connections between the right body of the hippocampus and other brain regions remained significant when controlling for GDS. Of these 85 relationships between rsFC strength and CCI score, 82 remained significant, 2 dropped to trend significance (*P* < 0.06) and 1 was no longer significant when controlling for GDS score. Lastly, exploratory analyses showed that GDS score was exclusively positively correlated with hippocampal rsFC strength, particularly between the left hippocampus and the rest of the brain (Supplementary Figure 1). Taken together, these analyses show that it is unlikely that our results related to CCI score are confounded by subclinical depressive symptoms.

## Discussion

Our results support smaller left hippocampal volume and weaker rsFC between the right body of the hippocampus and the DMN in cognitively normal individuals reporting SMD. The rsFC strength between the right body of the hippocampus and select brain networks, including those involved in memory, executive function and attention, was inversely associated with the severity of SMD, whereas hippocampal volume was not. This suggests that hippocampal rsFC patterns capture the current state of SMD in cognitively normal aged adults better than hippocampal volume, which may simply be a risk factor for SMD or may represent recent atrophy that is generally reflected in the current state of SMD (Cherbuin et al. 2015). Lastly, subclinical depressive symptoms were not associated with the above findings, and exploratory analyses showed that the severity of SMD and subclinical depressive symptoms are associated with different hippocampal connectivity patterns.

### Left Hippocampal Volume and Subfield Volumes are Smaller in SMD+

Our results showing decreased left whole hippocampal and left subfield volumes in SMD align well with the literature on SMD and more generally SCD, which may or may not specifically encompass SMD (van der Flier et al. 2004; Jessen et al. 2006; Saykin et al. 2006; Striepens et al. 2010; Scheef et al. 2012; Perrotin et al. 2015; Cantero et al. 2016; Ryu et al. 2017). Specifically, our results favoring involvement of the left hippocampus in SMD are reflected in the literature (van der Flier et al. 2004; Jessen et al. 2006; Striepens et al. 2010; Buckley et al. 2016; Cantero et al. 2016; Hu et al. 2019), and others have additionally noted the early vulnerability of the left hippocampus in AD (Thompson et al. 2004; Slavin et al. 2007; Shi et al. 2009). One recent study of hippocampal subfield volumes in cognitively normal individuals with SMD that used a highly similar, if not identical, automatic hippocampal subfield segmentation to the present study found smaller left molecular layer, CA1, CA4, dentate gyrus (DG) and whole hippocampal volumes in SMD (Cantero et al. 2016). Though our individual subfield results did not survive correction for multiple comparisons, we similarly found smaller left molecular layer, CA1, CA4, CA3, tail and whole hippocampal volumes in those with SMD. Left CA1 volume showed the strongest volume difference between groups, which aligns with the specific and important role of CA1 in human memory (Zola-Morgan et al. 1986; Bartsch et al. 2011). In line with this, Perrotin et al. (2015) found decreased CA1 volume in cognitively normal individuals reporting SCD. Both cognitively normal individuals with SCD and individuals with AD in that study showed decreased volume throughout CA1 when compared with cognitively normal individuals without SCD. Our findings support differences in left hippocampal structure between cognitively normal individuals with and without SMD.

### Weaker rsFC Strength between the right body of the hippocampus and the DMN in SMD+

Hippocampal rsFC strength was largely decreased in individuals reporting SMD. Weaker interactions between the right body of the hippocampus and the rest of the brain, specifically the DMN, were found in those reporting SMD. The DMN is a heavily studied brain network with relevance to aging and AD (Buckner et al. 2005; Hafkemeijer et al. 2012). Many have reported decreased rsFC between DMN regions in normal aging that is further decreased in the context of mild cognitive impairment (MCI) and AD (Dennis and Thompson 2014). The DMN has been associated with memory and self-referential processing, and stronger rsFC within the DMN has been associated with better memory function and other aspects of cognition (Andrews-Hanna et al. 2007; Sambataro et al. 2010; Wang et al. 2010; Ward et al. 2015; though see Jones et al. (2015) and Verfaillie et al. (2018) for both positive and inverse associations between DMN rsFC patterns and cognition). In line with these aspects of the DMN, we found that rsFC strength between the right body of the hippocampus and the DMN is weaker in individuals reporting SMD, which was also found by Wang et al. (2013) in an independent data set. Weaker rsFC between the hippocampus and DMN regions could thus reflect subtle memory changes that standard neuropsychological tests are unable to capture but that self-reported memory decline can capture. Our results align with the literature on the DMN in the context of aging and AD, the increased risk of pathological aging in those who report SMD and support a biological basis for SMD within memory systems.

The results of the studies that have investigated the DMN in the context of SMD and SCD are varied. As noted above, Wang et al. (2013) found decreased rsFC between the right hippocampus and the DMN in SCD, which was further decreased in amnestic MCI. Contreras et al. (2017) found that SCD severity was associated with decreased rsFC strength in several brain networks, including the DMN, across the AD spectrum. Viviano et al. (2019) recently reported decreased posterior memory system rsFC, which largely overlaps with the DMN, in cognitively normal individuals with SMD. Using magnetoencephalography, López-Sanz et al. (2017) found decreased rsFC strength within the posterior DMN in SCD, which was also present in individuals with MCI. In contrast, Hafkemeijer et al. (2013) exclusively found increased rsFC within the DMN, including between the hippocampus and other regions in the network, in SMD. Similarly, Verfaillie et al. (2018) recently reported increased rsFC strength between the posterior DMN and medial temporal memory system in SMD. Although we did not examine the rsFC strength between DMN regions, we found that rsFC strength between the right body of the hippocampus and specifically the DMN was reduced in SMD, suggesting greater isolation of this region of the hippocampus from the DMN in the state of SMD. Our analyses treating SMD as a continuous variable also supported this.

### The rsFC Strength between the right body of the hippocampus and Specific Brain Networks is Inversely Associated with the Severity of SMD

When we treated SMD as continuous, we found that hippocampal volume was not associated with it, but rsFC patterns were. The severity of SMD was strongly associated with the interactions between the right body of the hippocampus and specific brain networks, notably those involved in memory (DMN), executive function (COTCN, FPTCN) and attention (DAN), as well as those involved in sensorimotor function (AUD, SOM). The interpretation of the inverse relationship between the severity of SMD and rsFC strength between the right body of the hippocampus and the DMN is straightforward due to the strong association between the hippocampus and DMN with memory function and was discussed in the previous section. The inverse relationships between the severity of SMD and rsFC strength between the right body of the hippocampus and the other networks can be interpreted in 2 contexts that are not mutually exclusive. The first context is a functional model of SCD recently proposed by Viviano and Damoiseaux (2020). In this model, the authors propose that changes in the hippocampus and medial temporal lobe lead to disruptions in short-range, and notably, long-range connections between the DMN, executive control, and SNs. These disruptions (which would be reflected as weaker rsFC strength) then lead to memory encoding, memory retrieval, and executive functioning inefficiencies that cause the experience of SCD. Though we did not specifically examine rsFC patterns between these networks, our findings of weaker rsFC strength between the right body of the hippocampus and the DMN, cingulo-opercular task control and frontoparietal task control networks align with this model. Longitudinal studies that measure hippocampal volume, hippocampal activity during memory tasks, and rsFC patterns in SMD and SCD will be informative in continuing to investigate this model. The second interpretive context is the possibility that the individuals in this study were also experiencing subjective decline in other cognitive domains and that this decline is correlated with SMD. The inverse relationship between SMD severity and rsFC strength between the right body of the hippocampus and the cingulo-opercular task control, frontoparietal task control and dorsal attention networks might reflect subjective decline in executive function and attention. We did not assess subjective decline in executive function and attention in this study, but it is possible that subjective decline in these domains is correlated with SMD or that individuals perceive subjective decline in these domains as memory decline. Future studies that assess subjective decline in multiple cognitive domains will allow for an examination of the relationship between subjective decline in specific domains and rsFC strength in greater detail and would be informative.

The interactions between the right body of the hippocampus and other brain areas were closely associated with the degree of perceived memory decline at a given point in time. Left hippocampal volume was decreased in those reporting SMD though it was not associated with the strength of perceived decline at that same point in time. Smaller left hippocampal volume and subfield volumes (though these results did not survive correction for multiple comparisons) in individuals reporting SMD could represent the effects of processes that occurred earlier in the lifespan of these individuals that resulted in them being more susceptible to report SMD. Supporting this, a longitudinal study examining hippocampal atrophy in cognitively normal adults found that SMD at follow-up was associated with greater hippocampal atrophy over the prior 4-year period, but that SMD at baseline did not predict hippocampal atrophy over that same period (Cherbuin et al. 2015). It is also possible that being born with relatively smaller hippocampi is a risk factor for SMD later in life, though these explanations are speculative. Three recent studies that examined both rsFC patterns and structural patterns (gray matter volume, white matter tract integrity) in the same individuals found differential rsFC patterns associated with SMD but no differences in or associations with gray matter volume or white matter tract integrity and SMD (Yasuno et al. 2015; Kawagoe et al. 2019; Viviano et al. 2019), further supporting that rsFC patterns may be more sensitive to the current state of SMD than structural patterns. Longitudinal studies tracking changes in both features over time would be valuable to developing a better understanding of the structural and functional representation of SMD in the brain.

It is interesting that the volumetric findings associated with SMD were segregated to the left hippocampus and the rsFC findings associated with SMD were largely segregated to the right hippocampus. Although there is some support for these lateralized findings in the literature, the reason for this is unclear (Wang et al. 2013; Cantero et al. 2016; Hu et al. 2019). As discussed above, given that the left hippocampus and left subfield volumes were smaller in those with SMD but there was no correlation between left volumes and SMD severity, smaller left hippocampal and subfield volumes may be a risk factor for SMD. It is possible that the right hippocampus may have compensated for this at younger ages in these individuals. This compensation may have ultimately led to weaker rsFC strength between the right hippocampus and several brain networks, notably the DMN, in those with SMD. Though this is speculative, such a relationship between initial compensation followed by subsequent decline in functional connectivity strength has been discussed in the literature (Viviano and Damoiseaux 2020). Longitudinal studies incorporating volumetric and rsFC analyses of the hippocampus are required to test this hypothesis and will be informative in gaining a better understanding of this lateralization.

### Limitations

A few limitations of this study are important to note. The first is our use of T1W data alone to define hippocampal subfield volumes in FreeSurfer. It is possible that this contributed to the lack of relationship between SMD severity and hippocampal volumes, although the literature does not necessarily suggest that this relationship is present in cognitively normal individuals (though see Saykin et al. 2006). Regardless, future studies may find it beneficial to include a high-resolution T2W scan to help define the hippocampal data if possible. A second limitation is the cross-sectional nature of this study. Longitudinal studies are important to help us understand how hippocampal subfield volumes and rsFC patterns change in those reporting SMD and which features and changes best predict future pathological cognitive decline. Addressing these questions will provide a better understanding of the risk of pathological, objective cognitive decline in an individual reporting SMD and may also provide a better understanding of the role of self-awareness and self-referential cognition in this phenomenon. As the participants in the present study are followed over the next several years, the relationship between the measures evaluated in this paper and future cognitive decline can be examined. The third limitation is the lack of information on AD biomarker levels (amyloid and tau) in this sample. This would allow for independent assessment of which hippocampal features associated with SMD severity are most closely associated with AD risk as well as with amyloid and tau deposition. Both longitudinal and AD biomarker data would be valuable in connecting these findings more directly to AD risk. Without this information, our results nevertheless provide a valuable contribution to our understanding of how hippocampal interactions with specific brain networks are associated with the perception of memory decline in cognitively normal aged adults.

## Conclusion

Overall, our results show that hippocampal rsFC strength is more closely associated with current SMD severity than whole hippocampal or subfield volumes. Future work should continue the detailed, multimodal, and preferably longitudinal study of the hippocampus in the context of perceived memory decline in cognitively normal aged adults in order to better understand the development and representation of this state in the brain.

## Notes

The authors wish to thank the BUADC participants for dedicating their time to creating an important database that promises to advance our knowledge of aging through studies such as this one. *Conflict of Interest*: None declared.

## Funding

National Institutes of Health (grant P30 AG13846, K23AG046377, UL1TR001430).

## Supplementary Material

Zajac-Supplemental_Material_final_tgaa019

Zajac-SupplementalFigure1-final_tgaa019

## References

[ref1a] Aisen PS., Petersen RC, Donohue M, Weiner MW, & Alzheimer’s Disease Neuroimaging Initiative. (2015). Alzheimer’s disease neuroimaging initiative 2 clinical core: Progress and plans. Alzheimer’s & Dementia. 11(7): 734–739. 10.1016/j.jalz.2015.05.005 PMC4643840 26194309

[ref1] Amariglio RE , Mormino EC, Pietras AC, Marshall GA, Vannini P, Johnson KA, Sperling RA, Rentz DM. 2015. Subjective cognitive concerns, amyloid-beta, and neurodegeneration in clinically normal elderly. Neurology. 85:56–62. 26048028 10.1212/WNL.0000000000001712 PMC4501939

[ref2] Andrews-Hanna JR , Reidler JS, Sepulcre J, Poulin R, Buckner RL. 2010. Functional-anatomic fractionation of the brain's default network. Neuron. 65:550–562. 20188659 10.1016/j.neuron.2010.02.005 PMC2848443

[ref3] Andrews-Hanna JR , Snyder AZ, Vincent JL, Lustig C, Head D, Raichle ME, Buckner RL. 2007. Disruption of large-scale brain systems in advanced aging. Neuron. 56:924–935. 18054866 10.1016/j.neuron.2007.10.038 PMC2709284

[ref5] Barnes LL , Schneider JA, Boyle PA, Bienias JL, Bennett DA. 2006. Memory complaints are related to Alzheimer disease pathology in older persons. Neurology. 67:1581–1585. 17101887 10.1212/01.wnl.0000242734.16663.09 PMC2740723

[ref6] Bartsch T , Dohring J, Rohr A, Jansen O, Deuschl G. 2011. CA1 neurons in the human hippocampus are critical for autobiographical memory, mental time travel, and autonoetic consciousness. Proc Natl Acad Sci U S A. 108:17562–17567. 21987814 10.1073/pnas.1110266108 PMC3198338

[ref7] Buckley RF , Maruff P, Ames D, Bourgeat P, Martins RN, Masters CL, Rainey-Smith S, Lautenschlager N, Rowe CC, Savage G, et al. 2016. Subjective memory decline predicts greater rates of clinical progression in preclinical Alzheimer's disease. Alzheimers Dement. 12:796–804. 26852195 10.1016/j.jalz.2015.12.013

[ref8] Buckner RL , Snyder AZ, Shannon BJ, LaRossa G, Sachs R, Fotenos AF, Sheline YI, Klunk WE, Mathis CA, Morris JC, et al. 2005. Molecular, structural, and functional characterization of Alzheimer's disease: evidence for a relationship between default activity, amyloid, and memory. J Neurosci. 25:7709–7717. 16120771 10.1523/JNEUROSCI.2177-05.2005 PMC6725245

[ref9] Cantero JL , Iglesias JE, Van Leemput K, Atienza M. 2016. Regional hippocampal atrophy and higher levels of plasma amyloid-beta are associated with subjective memory complaints in nondemented elderly subjects. J Gerontol A Biol Sci Med Sci. 71:1210–1215. 26946100 10.1093/gerona/glw022 PMC4978360

[ref10] Cherbuin N , Sargent-Cox K, Easteal S, Sachdev P, Anstey KJ. 2015. Hippocampal atrophy is associated with subjective memory decline: the PATH through life study. Am J Geriatr Psychiatry. 23:446–455. 25204687 10.1016/j.jagp.2014.07.009

[ref11] Contreras JA , Avena-Koenigsberger A, Risacher SL, West JD, Tallman E, McDonald BC, Farlow MR, Apostolova LG, Goni J, Dzemidzic M, et al. 2019. Resting state network modularity along the prodromal late onset Alzheimer's disease continuum. NeuroImage: Clinical. 22:101687. 30710872 10.1016/j.nicl.2019.101687 PMC6357852

[ref12] Contreras JA , Goñi J, Risacher SL, Amico E, Yoder K, Dzemidzic M, West JD, McDonald BC, Farolw MR, Sporns O, et al. 2017. Cognitive complaints in older adults at risk for Alzheimer's disease are associated with altered resting-state networks. Alzheimers Dement (Amst). 6:40–49. 28149942 10.1016/j.dadm.2016.12.004 PMC5266473

[ref13] Dennis EL , Thompson PM. 2014. Functional brain connectivity using fMRI in aging and Alzheimer’s disease. Neuropsychol Rev. 24:49–62. 24562737 10.1007/s11065-014-9249-6 PMC4109887

[ref15] Filippini N , MacIntosh BJ, Hough MG, Goodwin GM, Frisoni GB, Smith SM, Matthews PM, Beckmann CF, Mackay CE. 2009. Distinct patterns of brain activity in young carriers of the APOE-epsilon4 allele. Proc Natl Acad Sci U S A. 106:7209–7214. 19357304 10.1073/pnas.0811879106 PMC2678478

[ref16] Galetta KM , Chapman KR, Essis MD, Alosco ML, Gillard D, Steinberg E, Dixon D, Martin B, Chaisson CE, Kowall NW, et al. 2017. Screening utility of the king-devick test in mild cognitive impairment and Alzheimer disease dementia. Alzheimer Dis Assoc Disord. 31:152–158. 27299935 10.1097/WAD.0000000000000157 PMC5154783

[ref17] Greene SJ , Killiany RJ. 2012. Hippocampal subregions are differentially affected in the progression to Alzheimer's disease. Anat Rec. 295:132–140. 10.1002/ar.21493 22095921

[ref18] Griffanti L , Salimi-Khorshidi G, Beckmann CF, Auerbach EJ, Douaud G, Sexton CE, Zsoldos E, Ebmeier KP, Flippini N, Mackay CE, et al. 2014. ICA-based artefact removal and accelerated fMRI acquisition for improved resting state network imaging. Neuroimage. 95:232–247. 24657355 10.1016/j.neuroimage.2014.03.034 PMC4154346

[ref19] Hafkemeijer A , Altmann-Schneider I, Oleksik AM, van de Wiel L, Middelkoop HA, van Buchem MA, van der Grond J, Rombouts SA. 2013. Increased functional connectivity and brain atrophy in elderly with subjective memory complaints. Brain Connect. 3:353–362. 23627661 10.1089/brain.2013.0144 PMC3749691

[ref20] Hafkemeijer A , van der Grond J, Rombouts SA. 2012. Imaging the default mode network in aging and dementia. Biochim Biophys Acta. 1822:431–441. 21807094 10.1016/j.bbadis.2011.07.008

[ref21] Hu X , Teunissen CE, Spottke A, Heneka MT, Düzel E, Peters O, Li S, Priller J, Buerger K, Teipel S, et al. 2019. Smaller medial temporal lobe volumes in individuals with subjective cognitive decline and biomarker evidence of Alzheimer's disease—data from three memory clinic studies. Alzheimers Dement. 15:185–193. 30321506 10.1016/j.jalz.2018.09.002

[ref22] Iglesias JE , Augustinack JC, Nguyen K, Player CM, Player A, Wright M, Roy N, Frosch MP, McKee AC, Wald LL, et al. 2015. A computational atlas of the hippocampal formation using ex vivo, ultra-high resolution MRI: application to adaptive segmentation of in vivo MRI. Neuroimage. 115:117–137. 25936807 10.1016/j.neuroimage.2015.04.042 PMC4461537

[ref23] Jack CR Jr , Petersen RC, O'Brien PC, Tangalos EG. 1992. MR-based hippocampal volumetry in the diagnosis of Alzheimer's disease. Neurology. 42:183–188. 1734300 10.1212/wnl.42.1.183

[ref24] Jack CR Jr , Petersen RC, Xu YC, O'Brien PC, Smith GE, Ivnik RJ, Boeve BF, Waring SC, Tangalos EG, Kokmen E. 1999. Prediction of AD with MRI-based hippocampal volume in mild cognitive impairment. Neurology. 52:1397–1403. 10227624 10.1212/wnl.52.7.1397 PMC2730146

[ref25] Jenkins A , Tales A, Tree J, Bayer A. 2015. Are we ready? The construct of subjective cognitive impairment and its utilization in clinical practice: a preliminary UK-based service evaluation. J Alzheimers Dis. 48:S25–S31. 26445273 10.3233/JAD-150541

[ref26] Jenkinson M , Beckmann CF, Behrens TE, Woolrich MW, Smith SM. 2012. Fsl Neuroimage. 62:782–790. 21979382 10.1016/j.neuroimage.2011.09.015

[ref27] Jessen F , Amariglio RE, van Boxtel M, Breteler M, Ceccaldi M, Chételat G, Dubois B, Dufouil C, Ellis KA, van der Flier WM, et al. 2014. A conceptual framework for research on subjective cognitive decline in preclinical Alzheimer's disease. Alzheimers Dement. 10:844–852. 24798886 10.1016/j.jalz.2014.01.001 PMC4317324

[ref28] Jessen F , Feyen L, Freymann K, Tepest R, Maier W, Heun R, Schild HH, Scheef L. 2006. Volume reduction of the entorhinal cortex in subjective memory impairment. Neurobiol Aging. 27:1751–1756. 16309795 10.1016/j.neurobiolaging.2005.10.010

[ref29] Jessen F , Wolfsgruber S, Wiese B, Bickel H, Mösch E, Kaduszkiewicz H, Petzek M, Riedel-Heller SG, Luck T, Fuchs A, et al. 2014. AD dementia risk in late MCI, in early MCI, and in subjective memory impairment. Alzheimers Dement. 10:76–83. 23375567 10.1016/j.jalz.2012.09.017

[ref30] Jones DT , Knopman DS, Gunter JL, Graff-Radford J, Vemuri P, Boeve BF, Petersen RC, Jack CR Jr, Alzheimer’s Disease Neuroimaging Initiative. 2015. Cascading network failure across the Alzheimer’s disease spectrum. Brain. 139:547–562. 26586695 10.1093/brain/awv338 PMC4805086

[ref1j] Jones DT, Machulda MM, Vemuri P, McDade EM, Zeng G, Senjem ML, Jack CR, Jr. 2011. Age-related changes in the default mode network are more advanced in alzheimer disease. Neurology. 77(16): 1524–1531. doi: 10.1212/WNL.0b013e318233b33d 10.1212/WNL.0b013e318233b33d PMC3198977 21975202

[ref31] Kawagoe T , Onoda K, Yamaguchi S. 2019. Subjective memory complaints are associated with altered resting-state functional connectivity but not structural atrophy. Neuroimage Clin. 21:101675. 30642761 10.1016/j.nicl.2019.101675 PMC6413342

[ref32] Kryscio RJ , Abner EL, Cooper GE, Fardo DW, Jicha GA, Nelson PT, Smith CD, Van Eldik LJ, Wan L, Schmitt FA. 2014. Self-reported memory complaints: implications from a longitudinal cohort with autopsies. Neurology. 83:1359–1365. 25253756 10.1212/WNL.0000000000000856 PMC4189103

[ref33] Krzywinski MI , Schein JE, Birol I, Connors J, Gascoyne R, Horsman D, Jones SJ, Marra MA. 2009. Circos: an information aesthetic for comparative genomics. Genome Res. 19:1639–1645. 19541911 10.1101/gr.092759.109 PMC2752132

[ref34] López-Sanz D , Bruña R, Garcés P, Martín-Buro MC, Walter S, Delgado ML, Montenegro M, López Higes R, Marcos A, Maestú F. 2017. Functional connectivity disruption in subjective cognitive decline and mild cognitive impairment: a common pattern of alterations. Front Aging Neurosci. 9:109. 28484387 10.3389/fnagi.2017.00109 PMC5399035

[ref35] Masters MC , Morris JC, Roe CM. 2015. "noncognitive" symptoms of early Alzheimer disease: a longitudinal analysis. Neurology. 84:617–622. 25589671 10.1212/WNL.0000000000001238 PMC4335988

[ref36] Mosconi L , De Santi S, Brys M, Tsui WH, Pirraglia E, Glodzik-Sobanska L, Rich KE, Switalski R, Mehta PD, Pratico D, et al. 2008. Hypometabolism and altered cerebrospinal fluid markers in normal apolipoprotein E E4 carriers with subjective memory complaints. Biol Psychiatry. 63:609–618. 17720148 10.1016/j.biopsych.2007.05.030 PMC2386268

[ref37] Mitchell A , Beaumont H, Ferguson D, Yadegarfar M, Stubbs B. 2014. Risk of dementia and mild cognitive impairment in older people with subjective memory complaints: meta-analysis. Acta Psychiatr Scand. 130:439–451. 25219393 10.1111/acps.12336

[ref38] Montejo P , Montenegro M, Fernández MA, Maestu F. 2011. Subjective memory complaints in the elderly: prevalence and influence of temporal orientation, depression and quality of life in a population-based study in the city of Madrid. Aging Ment Health. 15:85–96. 20924824 10.1080/13607863.2010.501062

[ref40] Norton DJ , Amariglio R, Protas H, Chen K, Aguirre-Acevedo DC, Pulsifer B, Castrillon G, Tirado V, Munoz C, Tariot P, et al. 2017. Subjective memory complaints in preclinical autosomal dominant Alzheimer disease. Neurology. 89:1464–1470. 28878053 10.1212/WNL.0000000000004533 PMC5631170

[ref41] Perrotin A , de Flores R, Lamberton F, Poisnel G, La Joie R, de la Sayette V, Mezenge F, Tomadesso C, Landeau B, Desgranges B, et al. 2015. Hippocampal subfield volumetry and 3D surface mapping in subjective cognitive decline. J Alzheimers Dis. 48:S141–S150. 26402076 10.3233/JAD-150087

[ref42] Power JD , Cohen AL, Nelson SM, Wig GS, Barnes KA, Church JA, Vogel AC, Laumann TO, Miezin FM, Schlaggar BL, et al. 2011. Functional network organization of the human brain. Neuron. 72:665–678. 22099467 10.1016/j.neuron.2011.09.006 PMC3222858

[ref43] Rabin LA , Smart CM, Amariglio RE. 2017. Subjective cognitive decline in preclinical Alzheimer's disease. Annu Rev Clin Psychol. 13:369–396. 28482688 10.1146/annurev-clinpsy-032816-045136

[ref44] Rattanabannakit C , Risacher L, Gao S, Lane KA, Brown SA, McDonald BC, Unverzagt FW, Apostolova LG, Saykin AJ, Farlow MR. 2016. The Cognitive Change Index as a measure of self and informant perception of cognitive decline: relation to neuropsychological tests. J Alzheimers Dis. 51:1145–1155. 26923008 10.3233/JAD-150729 PMC4833578

[ref45] Ryu SY , Lim EY, Na S, Shim YS, Cho JH, Yoon B, Hong YJ, Yang DW. 2017. Hippocampal and entorhinal structures in subjective memory impairment: a combined MRI volumetric and DTI study. Int Psychogeriatr. 29:785–792. 28067183 10.1017/S1041610216002349

[ref46] Salimi-Khorshidi G , Douaud G, Beckmann CF, Glasser MF, Griffanti L, Smith SM. 2014. Automatic denoising of functional MRI data: combining independent component analysis and hierarchical fusion of classifiers. Neuroimage. 90:449–468. 24389422 10.1016/j.neuroimage.2013.11.046 PMC4019210

[ref47] Sambataro F , Murty VP, Callicott JH, Tan H, Das S, Weinberger DR, Mattay VS. 2010. Age-related alterations in default mode network: impact on working memory performance. Neurobiol Aging. 31:839–852. 18674847 10.1016/j.neurobiolaging.2008.05.022 PMC2842461

[ref48] Saykin AJ , Wishart HA, Rabin LA, Santulli RB, Flashman LA, West JD, McHugh TL, Mamourian AC. 2006. Older adults with cognitive complaints show brain atrophy similar to that of amnestic MCI. Neurology. 67:834–842. 16966547 10.1212/01.wnl.0000234032.77541.a2 PMC3488276

[ref49] Scheef L , Spottke A, Daerr M, Joe A, Striepens N, Kolsch H, Popp J, Daamen M, Gorris D, Heneka MT, et al. 2012. Glucose metabolism, gray matter structure, and memory decline in subjective memory impairment. Neurology. 79:1332–1339. 22914828 10.1212/WNL.0b013e31826c1a8d

[ref50] Shi F , Liu B, Zhou Y, Yu C, Jiang T. 2009. Hippocampal volume and asymmetry in mild cognitive impairment and Alzheimer's disease: meta-analyses of MRI studies. Hippocampus. 19:1055–1064. 19309039 10.1002/hipo.20573

[ref51] Slavin MJ , Sandstrom CK, Tran TT, Doraiswamy PM, Petrella JR. 2007. Hippocampal volume and the mini-mental state examination in the diagnosis of amnestic mild cognitive impairment. Am J Roentgenol. 188:1404–1410. 17449789 10.2214/AJR.06.1052

[ref52] Sperling RA , Aisen PS, Beckett LA, Bennett DA, Craft S, Fagan AM, Iwatsubo T, Jack CR Jr, Kaye J, Montine TJ, et al. 2011a. Toward defining the preclinical stages of Alzheimer’s disease: recommendations from the national institute on aging-Alzheimer's association workgroups on diagnostic guidelines for Alzheimer's disease. Alzheimers Dement. 7:280–292. 21514248 10.1016/j.jalz.2011.03.003 PMC3220946

[ref53] Sperling RA , Jack CR Jr, Aisen PS. 2011b. Testing the right target and right drug at the right stage. Sci Transl Med. 3:111cm33. 10.1126/scitranslmed.3002609 PMC3752906 22133718

[ref54] Squire LR . 1992. Memory and the hippocampus: a synthesis from findings with rats, monkeys, and humans. Psychol Rev. 99:195. 1594723 10.1037/0033-295x.99.2.195

[ref55] Sripada C , Kessler D, Fang Y, Welsh RC, Prem Kumar K, Angstadt M. 2014. Disrupted network architecture of the resting brain in attention-deficit/hyperactivity disorder. Hum Brain Mapp. 35:4693–4705. 24668728 10.1002/hbm.22504 PMC6869736

[ref56] Striepens N , Scheef L, Wind A, Popp J, Spottke A, Cooper-Mahkorn D, Sulliman H, Wagner M, Schild HH, Jessen F. 2010. Volume loss of the medial temporal lobe structures in subjective memory impairment. Dement Geriatr Cogn Disord. 29:75–81. 20110703 10.1159/000264630

[ref57] Thompson PM , Hayashi KM, de Zubicaray GI, Janke AL, Rose SE, Semple J, Hong MS, Heran DH, Gravano D, Doddrell DM, et al. 2004. Mapping hippocampal and ventricular change in Alzheimer disease. Neuroimage. 22:1754–1766. 15275931 10.1016/j.neuroimage.2004.03.040

[ref58] Tsutsumimoto K , Makizako H, Doi T, Hotta R, Nakakubo S, Makino K, Shimada H, Suzuki T. 2017. Subjective memory complaints are associated with incident dementia in cognitively intact older people, but not in those with cognitive impairment: a 24-month prospective cohort study. Am J Geriatr Psychiatry. 25:607–616. 28216174 10.1016/j.jagp.2016.12.008

[ref59] van der Flier WM , van Buchem MA, Weverling-Rijnsburger AW, Mutsaers ER, Bollen EL, Admiraal-Behloul F, Westendorp RG, Middelkoop HA. 2004. Memory complaints in patients with normal cognition are associated with smaller hippocampal volumes. J Neurol. 251:671–675. 15311341 10.1007/s00415-004-0390-7

[ref60] Vannini P , Hanseeuw B, Munro CE, Amariglio RE, Marshall GA, Rentz DM, Pacual-Leone A, Johnson KA, Sperling RA. 2017. Hippocampal hypometabolism in older adults with memory complaints and increased amyloid burden. Neurology. 88:1759–1767. 28381517 10.1212/WNL.0000000000003889 PMC5409840

[ref61] Verfaillie SC , Binette AP, Vachon-Presseau E, Tabrizi S, Savard M, Bellec P, Ossenkoppele R, Scheltens P, van der Flier WM, JCS B, et al. 2018. Subjective cognitive decline is associated with altered default mode network connectivity in individuals with a family history of Alzheimer’s disease. Biol Psychiatry Cogn Neurosci Neuroimaging. 3:463–472. 29735156 10.1016/j.bpsc.2017.11.012

[ref62] Viviano RP , Damoiseaux JS. 2020. Functional neuroimaging in subjective cognitive decline: current status and a research path forward. Alzheimer's Res Ther. 12:1–18. 10.1186/s13195-020-00591-9 PMC7063727 32151277

[ref63] Viviano RP , Hayes JM, Pruitt PJ, Fernandez ZJ, van Rooden S, van der Grond J, Rombouts SARB, Damoiseaux JS. 2019. Aberrant memory system connectivity and working memory performance in subjective cognitive decline. Neuroimage. 185:556–564. 30308246 10.1016/j.neuroimage.2018.10.015

[ref64] Wang L , LaViolette P, O'Keefe K, Putcha D, Bakkour A, Van Dijk KR, Pihlajamaki M, Dickerson BC, Sperling RA. 2010. Intrinsic connectivity between the hippocampus and posteromedial cortex predicts memory performance in cognitively intact older individuals. Neuroimage. 51:910–917. 20188183 10.1016/j.neuroimage.2010.02.046 PMC2856812

[ref65] Wang Y , Risacher SL, West JD, McDonald BC, MaGee TR, Farlow MR, Gao S, O’Neill DP, Saykin AJ. 2013. Altered default mode network connectivity in older adults with cognitive complaints and amnestic mild cognitive impairment. J Alzheimers Dis. 35:751–760. 23481685 10.3233/JAD-130080 PMC3962306

[ref66] Ward AM , Mormino EC, Huijbers W, Schultz AP, Hedden T, Sperling RA. 2015. Relationships between default-mode network connectivity, medial temporal lobe structure, and age-related memory deficits. Neurobiol Aging. 36:265–272. 25113793 10.1016/j.neurobiolaging.2014.06.028 PMC4268379

[ref1w] White T, & Stern RA (2003). NAB, neuropsychological assessment battery: Demographically corrected norms manual. Psychological Assessment Resources.

[ref1ww] Whitfield-Gabrieli S, Nieto-Castanon A. (2012). Conn: A functional connectivity toolbox for correlated and anticorrelated brain networks. Brain Connectivity, 2(3), 125–141. 10.1089/brain.2012.0073 22642651

[ref67] Xia M , Wang J, He Y. 2013. BrainNet viewer: a network visualization tool for human brain connectomics. PLoS One. 8:e68910. 23861951 10.1371/journal.pone.0068910 PMC3701683

[ref68] Yasuno F , Kazui H, Yamamoto A, Morita N, Kajimoto K, Ihara M, Taguchi A, Matsuoka K, Kosaka J, Tanaka T, et al. 2015. Resting-state synchrony between the retrosplenial cortex and anterior medial cortical structures relates to memory complaints in subjective cognitive impairment. Neurobiol Aging. 36:2145–2152. 25862421 10.1016/j.neurobiolaging.2015.03.006

[ref69] Yekutieli D , Benjamini Y. 1999. Resampling-based false discovery rate controlling multiple test procedures for correlated test statistics. J Stat Plan Inference. 82:171–196.

[ref70] Zola-Morgan S , Squire LR, Amaral DG. 1986. Human amnesia and the medial temporal region: enduring memory impairment following a bilateral lesion limited to field CA1 of the hippocampus. J Neurosci. 6:2950–2967. 3760943 10.1523/JNEUROSCI.06-10-02950.1986 PMC6568782

