# Supplementary material for: Hippocampal Resting-State Functional Connectivity Patterns are More Closely Associated with Severity of Subjective Memory Decline than Whole Hippocampal and Subfield Volumes
Source: Cereb Cortex Commun. 2020 May 28;1(1):tgaa019. doi: 10.1093/texcom/tgaa019 (PMC7463163; doi:10.1093/texcom/tgaa019)
Supplement: Zajac-Supplemental_Material_final_tgaa019 [file zajac-supplemental_material_final_tgaa019.docx]

**Supplemental Material**

As an exploratory analysis, we examined what patterns of hippocampal functional connectivity were correlated with GDS score using the same approach used to carry out the CCI score analyses. In contrast to the relationship between hippocampal rsFC and CCI score, all 96 significant correlations between GDS score and hippocampal rsFC strength were positive. Of these 96 significant positive correlations, 48 involved the left head of the hippocampus and 26 involved the left body of the hippocampus, both above the false positive rate. Of the remaining significant positive correlations, 12 involved the right head of the hippocampus and 10 involved the right body of the hippocampus. Due to the large number of positive correlations between left hippocampal rsFC strength and GDS score, we tested whether the number of positive correlations between GDS score and rsFC strength between the left hippocampus (head and body) and other brain regions relative to the number of positive correlations involving the right hippocampus (head and body) was significantly greater than chance, and it was (p=0.0196). We also assessed whether there was network specificity among the connections between both left hippocampal regions and other brain regions that were positively correlated with GDS score. The number of significant positive correlations between GDS score and rsFC strength between the left hippocampus and the somatomotor (p=0.0296), memory retrieval (p=0.0298), visual (p=0.0241), and subcortical (p=0.0353) networks were significantly greater than chance. However, none of these patterns remained significant after adjusting for multiple comparisons.


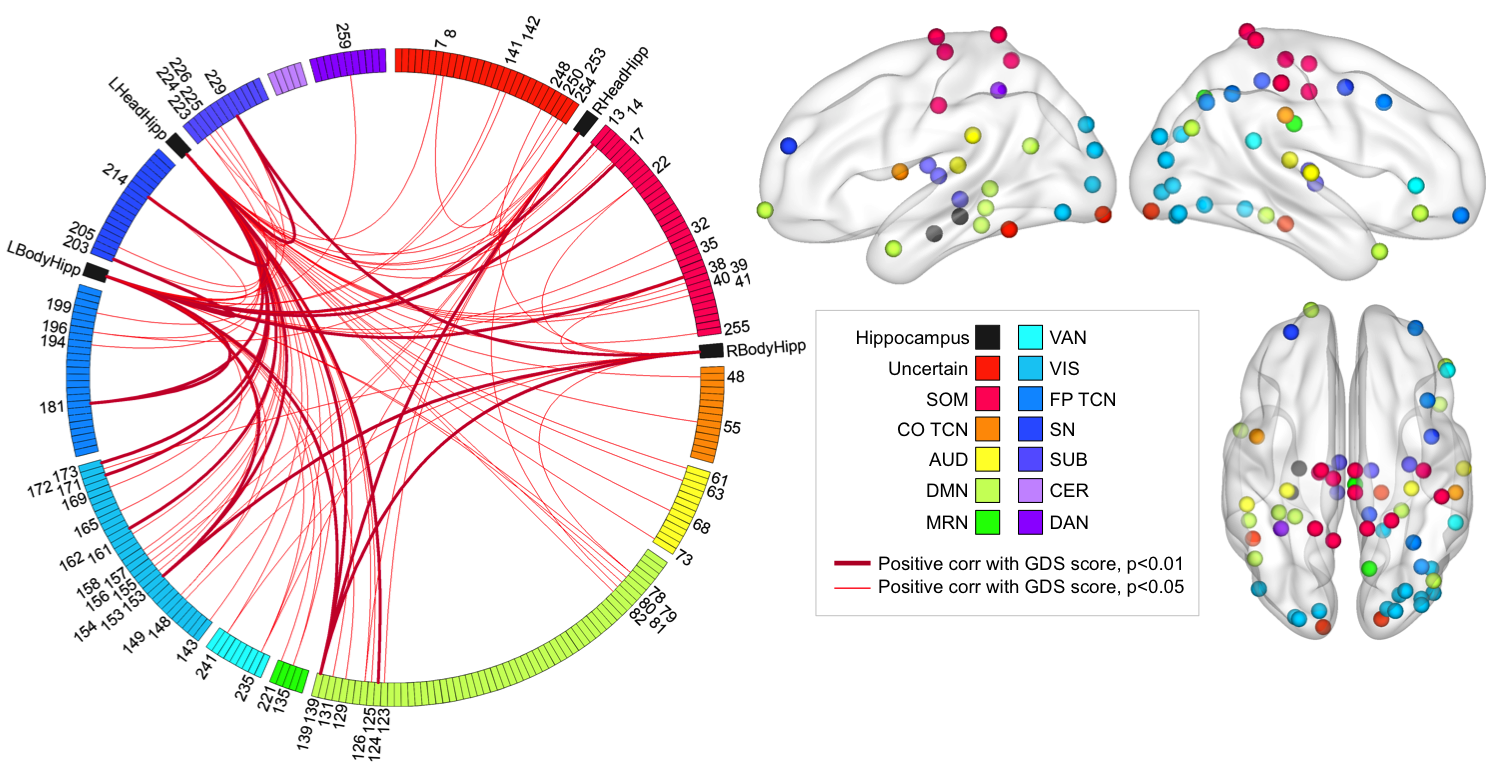


**Supplemental Figure 1: Hippocampal rsFC Strength is Positively Associated with Subclinical Depressive Symptoms.** The 96 connections between hippocampal and Power ROIs showing a positive relationship with GDS score are displayed on the Circos graph. No inverse relationships were found. The number of connections between the left hippocampus (head and body ROIs) and other brain regions showing an positive relationship with depressive symptoms relative to the number of connections between the right hippocampus (head and body ROIs) and the rest of the brain showing a positive relationship with depressive symptoms was greater than chance. The number of connections between the left hippocampus and specific brain networks showing positive relationships was not greater than chance for any network. The nodes representing the brain regions whose connection strength with the left hippocampus (head or body) showed a positive relationship with GDS score are displayed on the right on cortical surfaces created in Brain Net Viewer v1.6 (Xia et al., 2013). AUD: auditory network, CER: cerebellar network, CO TCN: cingulo-opercular task control network, DAN: dorsal attention network, DMN: default mode network, FP TCN: frontoparietal task control network, GDS: Geriatric Depression Scale (short form), LBodyHipp: left body of the hippocampus, LHeadHipp: left head of the hippocampus, MRN: memory retrieval network, RBodyHipp: right body of the hippocampus, RHeadHipp: right head of the hippocampus, ROI: region of interest, SN: salience network, SOM: somatomotor network, SUB: subcortical network, VAN: ventral attention network, VIS: visual network.

An association between subclinical depressive symptoms and SMD is frequently reported in the cognitively normal aged population and was found in our sample, as well. Earlier studies of SMD did not take these symptoms into account and it has since been recognized that it is important to do so moving forward (Jessen et al., 2014a). In our between-group analyses, when we accounted for response to the memory item on the GDS, no significant difference in GDS score was found between our SMD+ and SMD- groups, making it unlikely that the between-group findings are confounded by subclinical depressive symptoms. Because subclinical depressive symptoms were positively associated with SMD severity both when accounting for and not accounting for the memory item, we took care to ensure that our results treating SMD as a continuous variable were not confounded by these symptoms. Furthermore, our exploratory analysis showed positive associations between subclinical depressive symptoms and rsFC strength between the left hippocampus and the rest of the brain that was not network-specific, which lies in stark contrast to our results showing that SMD severity is inversely associated with rsFC strength between the right body of the hippocampus and specific brain networks. Subclinical depressive symptoms might be one factor that explains the mixed direction of rsFC findings in SMD in the literature and should continue to be investigated in the context of SMD in the cognitively normal aged population.
