# Supplementary figures and images for: Hippocampal Resting-State Functional Connectivity Patterns are More Closely Associated with Severity of Subjective Memory Decline than Whole Hippocampal and Subfield Volumes
Source: Cereb Cortex Commun. 2020 May 28;1(1):tgaa019. doi: 10.1093/texcom/tgaa019 (PMC7463163; doi:10.1093/texcom/tgaa019)

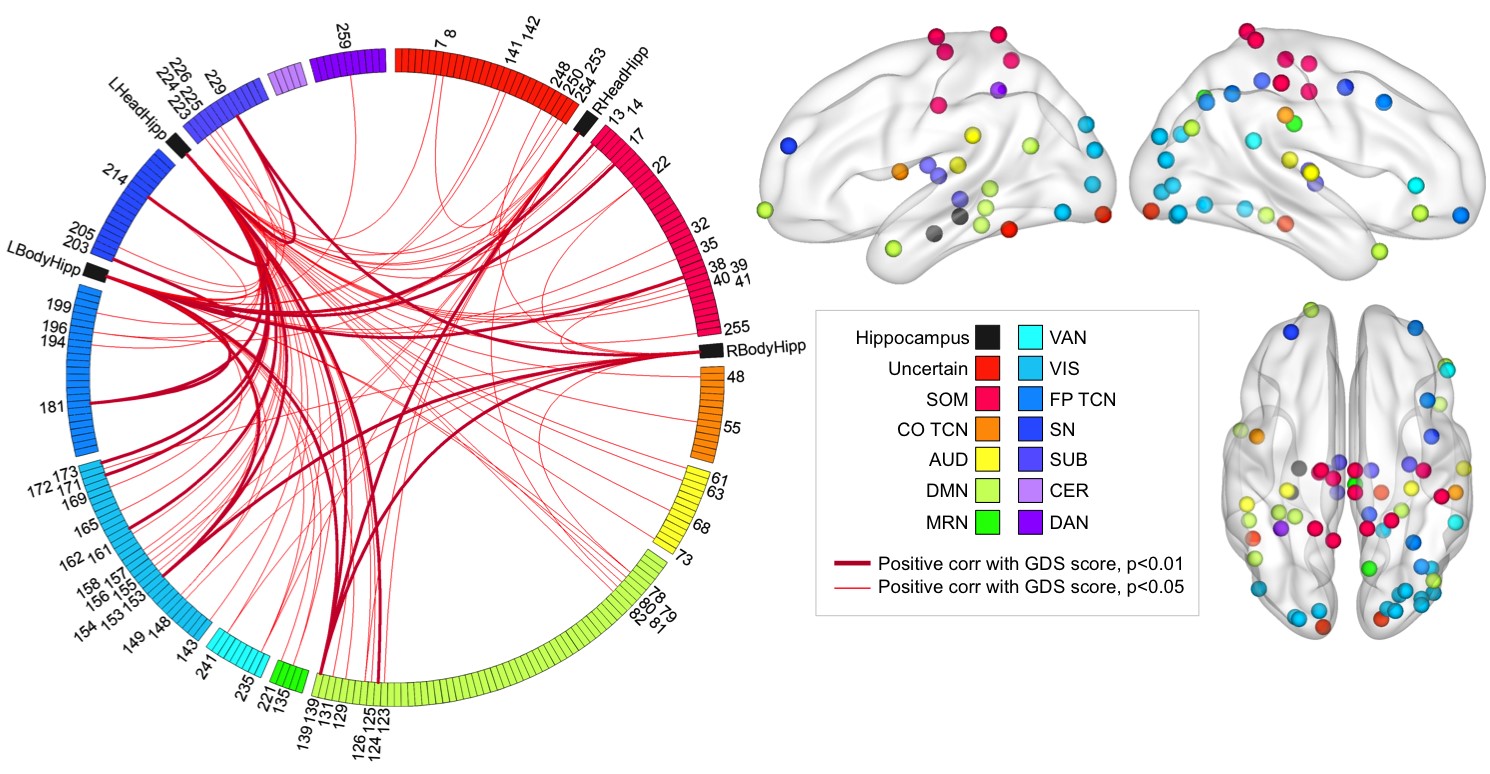

Supplement: Zajac-SupplementalFigure1-final_tgaa019 [file zajac-supplementalfigure1-final_tgaa019.jpeg]
